# Antimicrobial activity and properties of de novo design of short synthetic lipopeptides

**DOI:** 10.1007/s12223-024-01132-9

**Published:** 2024-01-26

**Authors:** Gabriela Kroneislová, Anna Macůrková, Zuzana Novotná, Rudolf Ježek, Petra Lovecká

**Affiliations:** 1 Department of Biochemistry and Microbiology, Faculty of Food and Biochemical Technology, University of Chemical Technology Prague, Prague, Czech Republic; 2 Department of Diary, Fat and Cosmetics, Faculty of Food and Biochemical Technology, University of Chemical Technology Prague, Prague, Czech Republic

**Keywords:** Antimicrobial peptides, De novo design, Structure–activity relationship, Solid phase peptide synthesis, Mechanism of action

## Abstract

The aim of this article is to introduce the topic of newly designed peptides as well as their biological activity. We designed nine encoded peptides composed of six amino acids. All these peptides were synthesized with C-terminal amidation. To investigate the importance of increased hydrophobicity at the amino end of the peptides, all of them were subsequently synthesized with palmitic or lithocholic acid at the N-terminus. Antimicrobial activity was tested on Gram-positive and Gram-negative bacteria and fungi. Cytotoxicity was measured on HepG2 and HEK 293 T cell cultures. Peptides bearing a hydrophobic group exhibited the best antimicrobial activity. Lipopeptides with palmitic or lithocholic acid (PAL or LCA peptides) at the N-terminus and with C-terminal amidation were highly active against Gram-positive bacteria, especially against strains of *Staphylococcus aureus* and *Candida tropicalis*. The LCA peptide SHP 1.3 with the sequence LCA-LVKRAG-NH_2_, had high efficiency on HepG2 human liver hepatocellular carcinoma cells (97%).

## Introduction

Nosocomial infections caused by multidrug-resistant pathogens are consistently a major topic in the medical and scientific community (Weinstein et al. 2005; Hidron et al. 2008). Globally, up to 700,000 deaths are due to infections caused by resistant bacteria every year. It is assumed that by 2050, this number will rise to 10 million, and these infections will become the most common cause of death (Review on Antimicrobial Resistance). Because of this ever-increasing antibiotic resistance, new possibilities are constantly being sought. One of them is antimicrobial peptides (AMP), which are an ancient part of innate immune mechanisms for controlling normal flora and fighting pathogens (Boman 1995; Andreu and Rivas 1998). They have been isolated from all species of animals and plants, and so far, more than two thousand sequences have been characterized in living organisms (Wang et al. 2009). Natural antimicrobial peptides (AMPs) are amphiphilic, combining cationic charges and hydrophobic components. It is well known that in many cases, AMPs form an α-helix structure with positive charges arrayed on one side and lipophilic groups aligned along the other side in contact with bacterial membranes. These small cationic peptides appear to act by the same mechanism as antibiotics, but many of them act through specific but non-receptor-mediated permeabilization of microbial membranes (Macurkova et al. 2014). All antimicrobial peptides interact with cell membranes (membrane disintegration or activity of the peptide at the intracellular level) (Doležílkova et al. 2011). The essential phase of the interaction of the peptide and the microorganism is the approach of AMP to the cell membrane, mediated by the electrostatic interaction between the peptide and the membrane surface, in Gram-negative bacteria with lipopolysaccharides and phospholipids, and in Gram-positive bacteria with teichoic acid (Brogden 2005). They can fold into various secondary structures, such as α-helices and β-sheets, or they can form cycles and hairpin loops. Despite their diversity, most AMPs share common features, including positive charge and amphipathic character (Hancock 1999; Shai 2002). Some AMPs not only exhibit affinity to prokaryotic cells, but also to higher eukaryotic cells (Hwang and Vogel 1998). Structure–activity relationship studies of these peptides suggest that changes in amphiphilicity could be used to separate antimicrobial activity from hemolytic activity and toxicity (Kondejewski et al. 1996). Bile acids are cholesterol-derived amphiphilic steroid acids produced in mammals and other vertebrates. There are four different derivatives of bile acids, which vary by the number of hydroxyl groups, such as cholic acid (CA), deoxycholic acid (DCA), chenodeoxycholic acid, and lithocholic acid (LCA). The steroidal nucleus with four fused rings provides a hydrophobic core with a significantly larger cross-sectional area compared to linear alkyl chains. The facial amphiphilicity, biocompatibility, and hydrophobicity of bile acid derivatives are considered to be highly favorable for interactions with bacterial cell membranes (Lin et al. 2021). In this study, we present de novo-designed synthetic cationic peptides and their lipopeptide conjugates with palmitic or lithocholic acid at the N-terminus (PAL/LCA lipopeptide). We designed three encoded hexapeptides (SHP), one according to the template (Tossi et al. 1997) and the other two with a random sequence, which were amphipathic in character and had a positive charge. We increased the lipophilicity of SHPs by conjugation with palmitic acid or lithocholic acid. The design of peptides was mainly based on the relative frequency of amino acids in natural antimicrobial peptides with the required properties, such as length, activity, or conformation. These peptides were designed according to the already known peptide isolated from coconut water (Mandal et al. 2009), with a sequence of up to 10 amino acids exhibiting an antimicrobial effect. This selection was based on statistical data obtained from databases (Wang et al. 2009; Tossi et al. 1997). Helical peptides are generally more active; therefore, the amino acid sequence was assembled to form a peptide with a helical conformation. Many sources have shown that C-terminal amidation (Cabrera et al. 2008) or N-terminal acylation increases activity (Radzishevsky et al. 2005). Our study was also focused on the description of the biological properties, mainly antimicrobial and hemolytic activity, or cytotoxicity of short cationic peptides and their lipopeptide conjugates.

## Material and methods

The chemicals benzotriazol-1-yl-oxytripyrrolidinophosphonium hexafluorophosphate (PyBOP), Rink amide MBHA resin (100–200 mesh), and triisopropylsilane (TIS) were purchased from Novabiochem (Darmstadt, DEU); N,N-diisopropylethylamine (DIEA) and the Mueller–Hinton agar with sheep blood were obtained from Merck (Darmstadt, DEU); 9-fluorenylmethoxycarbonyl (Fmoc)-protected amino acids: Fmoc-Ala-OHxH2O, Fmoc-Gly-OH, Fmoc-Lys(Boc)-OH (Boc-t-butyloxycarbonyl), Fmoc-L-Leu-OH, Fmoc-L-Arg(Pbf)-OH (Pbf = 2,2,4,6,7-pentamethyldihydrobenzofuran-5-sulfonyl), Fmoc-L-Val-OH, trifluoroacetic acid (TFA), N,N’-diisopropylcarbodiimide (DIC), and piperidine (PIP) were obtained from Iris Biotech (Marktredwitz, DEU); 1-hydroxybenzotriazole (HOBt), 2-aminofluorene, daunomycin, glucose-6-phosphate, NADP, lithocholic acid (LCA), palmitic acid (PAL), Luria–Bertani’s medium, Mueller–Hinton’s broth 2, Mueller–Hinton’s agar, malt extract broth, amphotericin B (AMF), ampicillin (AMPI), clotrimazole (CLO), erythromycin (ERY), kanamycin (KAN), tetracycline (TET), and vancomycin (VAN) were purchased from Sigma-Aldrich (St. Louis, MO, USA); RPMI medium with FBS (10%, fetal bovine serum) and MEM vitamin solution (1%) were obtained from Invitrogen (Carlsbad, CA, USA); the Cytotoxicity Detection Kit (LDH) was obtained from Roche (Mannheim, DEU); high-performance-liquid-chromatography (HPLC) grade acetonitrile (ACN), isopropyl alcohol (IPA), dichloromethane (DCM), and dimethylformamide (DMF) were obtained from Lachner (Neratovice, CZE); the QIAprep^®^ Spin Miniprep Kit was from Qiagen GmbH (Hilden, DEU); restriction endonuclease EcoRV was from New England Biolabs Inc. (Ipswich, MA, USA); 5xTris borate-EDTA buffer was from 5 PRIME Inc. (Gaithersburg, MD, USA); agarose was from Serva Electrophoresis GmbH (Heidelberg, DEU).

## Peptide design

Six amino acids were chosen from databases (Wang et al. 2009) on the basis of their frequency of occurrence in natural antimicrobial peptides with the required properties, such as a helical conformation and activity against both Gram-positive and Gram-negative bacteria, fungi, and cancer cells. The presence of positively charged amino acids was the other requirement. Alanine, glycine, leucine, lysine, arginine, and valine were chosen as the best candidates for the rational design of these peptides. The position of individual amino acids in the sequence was determined by the relative frequency of six different types of residues in the first 20 positions of natural α-helical antimicrobial peptides (Tossi et al. 1997). C-terminal amidation (Cabrera et al. 2008; Meylaers et al. 2003; Grif et al. 1998) or modification with a fatty acid (palmitic acid; PA or lithocholic acid; LCA) at the N-terminus (Radzishevsky et al. 2005) was monitored according to their ability to increase antimicrobial activity. In every step of the peptide design, biophysical properties such as molecular weight, total net charge, isoelectric point (http://www.chemaxon.com/marvin/sketch/index.php), hydrophobic moment (http://www.bbcm.univ.trieste.it/~tossi/HydroCalc/HydroMCalc.htmL), and helical wheel projection (http://cti.itc.virginia.edu/~cmg/Demo/wheel/wheelApp.htmL) were calculated. Peptide structures were studied by the method of parallel tempering. The system containing the peptide, three chloride anions, and 852–854 water molecules was simulated in forty replications at temperatures from 280 to 500 K (6.85 to 226.85 °C). Every 2 ps coordinates could be swapped between neighboring replications. Each peptide was simulated for 40 × 10 ns. The trajectory at 301.6 K (28.45 °C) was analyzed using the essential dynamics method, and the free energy surface was calculated (software Gromacs).

## Synthesis of peptides with C-terminal amidation and N-terminal acylation

The designed peptides were prepared by solid-phase peptide synthesis (SPPS), which proceeds from the C-terminus to the N-terminus of the peptide. Rink resin (*S* = 1.1 mmol/g for peptide-amides) was used as the solid phase. This type of synthesis uses N-protected amino acids, which include the protecting group Fmoc (fluorenylmethyloxycarbonyl). The synthesis consists of several repetitive steps. First of all, load the first amino acids according to the Melm/MSNT method in DCM for 4 h. These steps are repeated until all the required amino acids are bound in the peptide. Then, all protecting groups of the side chains are removed, and the entire peptide is cleaved from the resin. The general procedure for each synthetic cycle, based on 200 mg of initial Rink resin, was as follows: (1) deprotection of Fmoc – 20% PIP in DMF, ≥ 20 min; (2) washing with DMF, 3 × 1 min, IPA, 3 × 1 min, DMF, 3 × 1 min; (3) amino acid coupling – HOBt, 5 eq in DMF, Fmoc-AA, 4 eq in DMF, 2 M DIC/DMF, 7 eq, 15 µL 1‰ bromophenol blue (BB) in DMF, shaking in the reaction vessel at room temperature for at least 2 h; the reaction time was corrected on the basis of indicator color; (4) washing with DMF, 3 × 1 min, IPA, 3 × 1 min, DMF, 3 × 1 min. The binding of palmitic (PAL) or lithocholic acid (LCA) was performed on the N-terminus of the peptide on the resin in the same way as previous amino acids with two recouplings, the former with HOBt, 5 eq in DMF, Fmoc-AA, 4 eq in DMF, 2 M DIC/DMF, 7 eq, for 2 h and the latter one using PyBOP, 4.9 eq in DMF, acid, 5 eq in DMF, DIEA, 10 eq for 3 h. The cleavage of (PAL/LCA)-peptides from the resin and deprotection of the side chains were performed in a solution of 95% TFA, 2.5% TIS, and 2.5% H_2_O, and the reaction time was 3.5 h, followed by 2–3 TFA washings. TFA was removed with a stream of nitrogen. PAL/LCA-peptides with C-terminal amidation were precipitated with t-butylmethylether and collected by centrifugation (5000 × g, 2 min).

## Analytical assay of peptides with C-terminal amidation and N-terminal acylation

After the synthesis, the PAL/LCA-peptides were purified by RP-HPLC (HP 1100, Agilent, USA) in a Discovery BIO Wide Pore C8 column (250 × 10 mm; 5 μm particle size), (Sigma-Aldrich, St. Louis, MO, USA) using a 100-min gradient from 0 to 100% ACN (3 mL/min). Gradient elution was performed with a solution of increasing concentration of acetonitrile in deionized water with 0.1% trifluoroacetic acid. The absorbance of the eluate was measured at 220 and 280 nm. Samples were taken at mid-elution times and analyzed by MALDI-TOF MS using MALDI-TOF Autoflex (Bruker Daltonics, Billerica, MA, USA).

## Biological assay

### Agar dilution test

The agar dilution test was performed for a qualitative determination of antimicrobial activity. Petri dishes with 20 mL of solid Mueller–Hinton agar for bacteria or Malt extract agar for fungi were overlayered with 3 mL of semisolid agar (0.5 g of agar per 100 mL of adequate liquid growth medium) with 100 µL of microbial culture grown overnight (dilution to OD_600_ 0.1). The volume of the tested compounds was 2 µL, and their concentration was 10 mg/mL. Zones determining antimicrobial activity were detected after 24 h of incubation at an appropriate temperature.

### Quantitative determination of antimicrobial activity

A Bioscreen C automated microbiology growth curve analysis system (Growth Curves, Piscataway, NJ, USA) was used for the quantitative determination of antimicrobial activity (minimal inhibitory concentration, MIC) of PAL/LCA-peptides. Optical density was measured with a wide bandpass filter (wavelength range 420–580 nm) at regular intervals and temperatures. This assay was performed in a special microtiter plate (10 × 10 wells) that allowed the simultaneous testing of a large number of samples against several microorganisms. The total testing volume was 100 µL, obtained by mixing 10 µL of microbial culture with OD600 equal to 0.1 with growth medium and a solution of the tested compounds. The positive controls were vancomycin and tetracycline for *Bacillus subtilis* (100 µg/mL) for Gram-positive bacteria, kanamycin, and tetracycline for *Acinetobacter baumannii* (100 µg/mL) for Gram-negative bacteria, and clotrimazole or amphotericin (100 µg/mL) for fungi. The maximal concentration of tested lipoproteins was 1 mg/mL. A pure growth medium was used as the negative control. The measurement period was 25 h. The lowest concentration with a 100% inhibition effect for 24 h of incubation was chosen as the MIC. The antibacterial activity of each compound was expressed as the minimum inhibitory concentration MIC (MIC is the lowest concentration of the compound needed to inhibit the growth of bacterial cells) and IC_50_ (IC_50_ is the concentration of the compound needed to inhibit the growth of bacterial cells by 50%).

### Mechanism of action

#### Killing kinetics

This assay was used to determine how quick the effect of PAL/LCA-peptides is on bacteria, as well as whether the activity of these compounds is dependent on the presence of active bacterial metabolism (Kamysz et al. 2007). The bacterial cultures were incubated overnight at 37 °C in Mueller–Hinton’s broth 2 or potassium phosphate buffer (0.1 M, pH 7). Cultures were diluted to OD_600_ equal to 0.1 and divided into 2 samples, and the final volume of each sample was 1 mL; after that, they were centrifuged (5000 × g, 10 min) to collect bacteria. The supernatant was discarded, and cells were resuspended in Mueller–Hinton’s broth 2 (the former parallel) or potassium phosphate buffer (0.1 M, pH 7) (the latter parallel), with (PAL/LCA)-peptide at a concentration equal to 2xMIC value for the tested bacteria; the control samples were free of the tested compounds. The samples were incubated at 37 °C. At specific time intervals, samples of bacterial suspension were taken, diluted, and spread on Petri dishes with Mueller–Hinton’s agar to count the number of surviving cells as colony-forming units (CFU). The whole experiment lasted 24 h.

#### Membrane disruption

The disruption of the microbial membrane was followed by the detection of lactate dehydrogenase activity released from the intracellular space using a Cytotoxicity Detection Kit (LDH) from Roche (Korzeniewski and Callewaert 1983; Macurkova et al. 2014) after 1 h incubation at 37 °C with the PAL/LCA-peptide. The culture supernatant was collected and incubated with the reaction mixture from the kit. In the first step, NAD^+^ is reduced to NADH + H^+^ by the LDH-catalyzed conversion of lactate to pyruvate. In the second step, the catalyst (diaphorase) transfers H/H^+^ from NADH + H^+^ to the tetrazolium salt 2-(4-iodophenyl)-3-(4-nitrophenyl)-5-phenyl-2H-tetrazolium chloride (INT), which is reduced to formazan. The formazan dye formed is red and water-soluble and gives a broad absorption maximum at 500 nm. An increase in the amount of dead or plasma membrane-damaged cells results in an increase in the LDH activity in the cell culture supernatant.

To determine the degree of damage to the cell membrane (%) by the tested substances, averages of absorbances (triplets) were calculated from the measured values for the samples, controls, and pure medium. Subsequently, the absorbance average for the clean medium (background) was subtracted from all averages obtained in this way. The degree of damage to the cell membrane by the tested substances was expressed as follows:

$$\mathrm{membrane \;damage }\left(\mathrm{\%}\right)=((AVZ-\mathrm{\varnothing }ANK)/(\mathrm{\varnothing }APK-ANK))\bullet 100$$

$$\varnothing \mathrm{AVZ}\dots \mathrm{average \;absorbance \;of \;sample}$$

$$\varnothing\mathrm{ANK}\dots \mathrm{average \;absorbance \;of \;negative \;control}$$

$$\varnothing \mathrm{APK}\dots \mathrm{average \;absorbance \;of \;positive \;control}$$

## DNA binding assay

This assay was performed as described by Park et al. (1998), with a few modifications. Fifty nanograms of circular or linearized plasmid DNA (pBluescriptII KS-(QIAprep^®^ Spin Miniprep Kit, Qiagen)) was mixed with an increasing amount of (PAL/LCA)-peptides in 20 µL of binding buffer (5% glycerol, 10 mM Tris–HCl (pH 8.0), 1 mM EDTA, 1 mM DTT, 20 mM KCl and 50 µg/mL BSA). The reaction mixture was incubated at room temperature for 1 h. Subsequently, 4 µL of native loading buffer (10% Ficol, 10 mM Tris–HCl (pH 7.5), 50 mM EDTA, 0.25% bromophenol blue) was added. An aliquot was applied to 1% agarose gel electrophoresis in 0.5xTris borate-EDTA buffer. Circular plasmid DNA was isolated from (Luria–Bertani medium with AMPI in final concentration 100 µg/mL) bacterial strain *E. coli* DH5α / pBluescriptII KS- that had been grown overnight, and linearization was performed overnight by enzymatic reaction using restriction endonuclease EcoRV at 37 °C. The evaluation was performed by visualizing the DNA with the intercalation dye Midori Green Advanced DNA Stain under UV light. Reduced electrophoretic mobility compared to the control is considered to be evidence of peptide binding to DNA.

## Transmission electron microscopy

Pictures of damaged bacterial cells were obtained by transmission electron microscopy after 1 to 4 h of incubation. Cells of the Gram-positive bacterial strains were not stained. A JEOL JEM-1010 transmission electron microscope (JEOL, Tokyo, Japan) equipped with a MegaView III CCD camera was used for visualization, and evaluation was performed with the program for image analysis AnalySIS software v. 2.0 (Soft Imaging Systems). The samples were plated on a carbon-coated electron microscopic grid and observed with an accelerating voltage of 80 kV.

## Toxicity assay

### Hemolytic activity assay

Hemolytic activity assay was performed by the detection of zones of hemolytic activity formed after 24 h on Mueller–Hinton’s agar with sheep blood (Macurkova et al. 2014). The tested concentration range of each (PAL/LCA)-peptide was identical to the previous antimicrobial assay, and the tested volume was 2 µL. Hemolytic antimicrobial peptide chrysophsin-3 (Iijima et al. 2003) was used as the positive control and solvent (deionized water) as the negative control.

### Cytotoxicity assay

Experiments were carried out using an xCELLigence RTCA DP Instrument (Roche Diagnostics GmbH, Mannheim, Germany) which was placed into an incubator (37 °C and 5% CO_2_). Cell proliferation and cytotoxicity experiments were performed using modified 16-well plates (E-plate, Roche Diagnostics GmbH, Mannheim, Germany). Microelectrodes were attached to the bottom of the wells for the impedance-based detection of attachment, spreading, and proliferation of the cells (Stoddart 2011). This system monitors cellular events in real time without the incorporation of labels by measuring electrical impedance across an interdigitated microelectrode integrated into the bottom of this special tissue culture plate. This impedance measurement improves upon conventional endpoint assays and provides quantitative information about the biological status of the cells, including cell number, adhesion, viability, and morphology. Ninety microliters of cultivation medium was used for the blank sample, and subsequently, 100 µL of HepG2 or HEK293T cell suspension (7 × 10^5^ cells/mL) was added and placed into the incubator (37 °C, 95% humidity, 5% CO_2_). After 24 h, 10 µl of solution of the tested (PAL/LCA)-peptide was added into the suspension with adherent cells. Chrysophsin-3 at a concentration of 100 µg/mL was used as a positive control and solvent (deionized water) as a negative control. Subsequent incubation was performed for at least an additional 24 h. The result of the measurement was viability curves. After the measurement, percentages of tissue culture growth inhibition were calculated for all the tested substances, as follows:

$$\%\;\mathrm{inhibition}\;=(1-(\Delta\phi CI\mathrm{sample})/(\Delta\phi CI\mathrm{control}))\cdot100$$

Δ∅CIsample … the difference between the first and last average CI (cell index) values of the tissue culture in the presence of the tested sample.

Δ∅CIcontrol … the difference between the first and last average CI values of the tissue culture itself.

## Results

### Peptide preparation

**Fig. 1:**
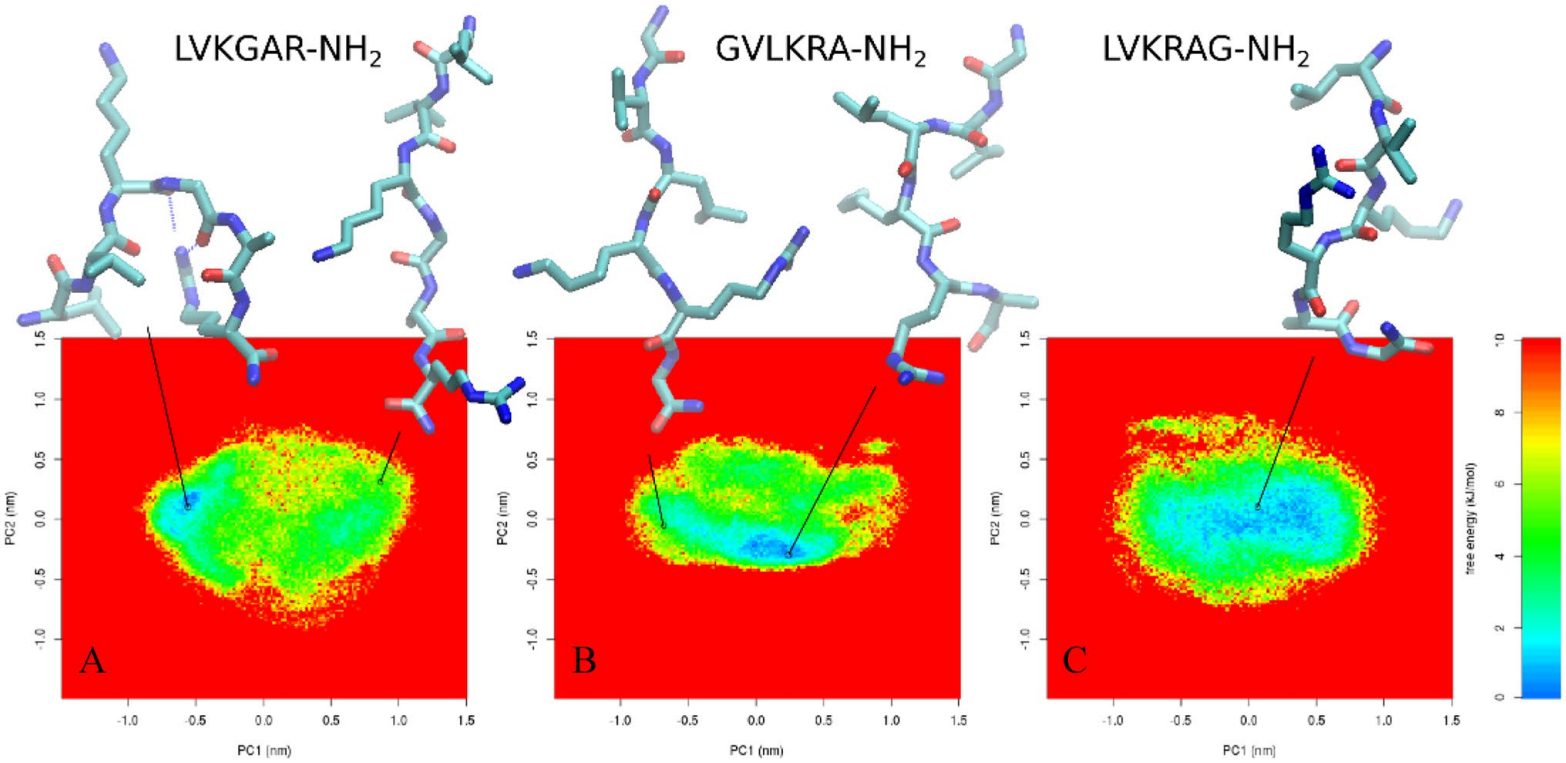
Molecular model of primary peptide SHP-1.1 (**A**), SHP-2.1 (**B**), and SHP-3.1 (**C**)

**Fig. 2:**
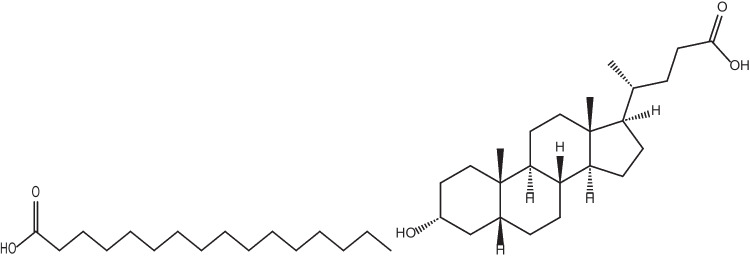
Structure of palmitic acid (left) and lithocholic acid (right)

Natural AMPs from different sources exhibit a variety of post-translational modifications including glycosylation, methylation, and acylation. The C-terminal amidation of a peptide generally enhances its antimicrobial activity, decreases its cytotoxicity, and associates with its resistance to enzymatic degradation. C-terminal amidation alters two key properties simultaneously: the net charge and helicity of the peptide, both of which are implicated in the mechanism of action. It has been proposed that the enhanced antimicrobial activity exhibited by the amidated forms is associated with a more rigid and extended alpha-helical structure. An amidated C-terminal end produces an increase in net positive charge by + 1 by masking the otherwise anionic C-terminal carboxylate group. We, therefore, only synthesized peptides with C-terminal amidation. On the basis of the calculated data, 3 primary amino acid sequences which fulfilled our requirements (SHP-1.1 – LVKRAG-NH_2_, SHP-2.1 – LVKGAR-NH_2,_ and SHP-3.1 – GVLKRA-NH_2_) were chosen and prepared. Alanine, glycine, leucine, lysine, arginine, and valine were chosen as the best candidates for the rational design of peptides. These primary peptides exhibited no significant similarity with existing natural antimicrobial peptides, and they had a positive charge and were able to adopt a helical structure, which was calculated as the hydrophobic moment (Hand 2000; Levy 1998). Subsequently, their probable secondary structure was predicted for the proposed sequences by molecular modeling (Fig. 1). All peptides were synthesized from L-amino acids. Lipopeptide conjugates—lipophilic derivatives of short peptide amides—were prepared by acylation of the N-terminus of peptide with palmitic acid or lithocholic acid (Fig. 2). The derivatives of primary peptides were designed and synthesized in the same way as basic peptides, i.e., by acylation of the N-terminus of a peptide with palmitic (PAL-, fatty) (SHP-1.2 – PAL-LVKRAG-NH_2_, SHP-2–2 – PAL-LVKGAR-NH_2_, and SHP-3.2 – PAL-GVLKRA-NH_2_) or lithocholic (LCA-, bile) acid (SHP-1.3 – LCA-LVKRAG-NH_2_, SHP-2.3 – LCA-LVKGAR-NH_2_, and SHP-3.3 – LCA-GVLKRA-NH_2_). We used lipophilic chains because of the possibility of increasing their antimicrobial activity (easier penetration into the cell) (Brogden 2005; Radzishevsky et al. 2005).

## Purification and analysis of primary peptides and lipopeptides with C-terminal amidation

After the synthesis, short cationic peptides and their lipopeptide conjugates with C-terminal amidation were purified with a C8 reversed-phase high-performance liquid chromatography (RP-HPLC) column. Gradient elution was performed with a solution of increasing concentration of acetonitrile in deionized water with 0.1% trifluoroacetic acid. The absorbance of the eluate was measured at 220 and 280 nm. Elution zones with a significant response were analyzed by MALDI-TOF–MS. Elution zones corresponding to the desired peptides and lipopeptides were identified based on mass analysis. MALDI-TOF MS (Bruker Daltonics) was used to identify the proper fractions with the target peptide. MALDI-TOF mass spectrometry analyses were carried out with an Autoflex Speed mass spectrometer (Bruker Daltonics, Germany) equipped with a UV nitrogen laser (337 nm)/ Nd:YAG laser (355 nm). The samples of peptides were prepared by mixing 1 µl of peptide solution and 9 µL of freshly prepared matrix solution (15 mg/mL of 2,5-dihydroxybenzoic acid in 1:2 acetonitile:0.1% TFA). A total of 1 µL of this mixture was placed on a stainless steel probe plate and allowed to dry at room temperature. The spectra were recorded in positive reflector mode in the range from 500 to 4000 Da. Characteristics of synthetic peptides are listed in Table 1.

**Table 1:** Chromatographic and mass characteristics of synthetic primary peptides and lipopeptides

Sequence	MWc (Da)^a^	MWm (Da)^b^	Ret. time (min)
SHP-1.1LV**KR**AG-NH_2_	642.8	642.973	15.160
SHP-1.2PAL-LV**KR**AG-NH_2_	880.6	880.632	22.851
SHP-1.3LCA-LV**KR**AG-NH_2_	1000.8	1000.842	24.751
SHP-2.1LV**K**GA**R**-NH_2_	642.8	642.823	8.701
SHP-2.2PAL-LV**K**GA**R**-NH_2_	880.6	880.751	22.325
SHP-2.3LCA-LV**K**GA**R**-NH_2_	1000.8	1000.716	23.005
SHP-3.1**GV**L**KR**A-NH_2_	642.8	642.712	4.464
SHP-3.2PAL-**GV**L**KR**A-NH_2_	880.6	880.718	22.576
SHP-3.3LCA-**GV**L**KR**A-NH_2_	1000.8	1000.830	24.300

^a^*MWc*, calculated molecular weight

^b^*MWm*, measured molecular weight

## Structure: activity relationship study

In this study, compounds were synthesized to investigate whether a change in the position of the cationic groups of peptides and N-terminal acylation would influence their antimicrobial activities. All of the designed and subsequently prepared PAL/LCA-peptides were screened for activity against a wide range of microorganisms, including Gram-positive and Gram-negative bacteria and fungi. The microorganisms were chosen on the basis of their distribution around the world and their ability to acquire resistance to antibiotics (Hand 2000; Levy 1998). Agar diffusion assay was utilized for the initial screening of activity, and after positive results, the microtitre dilution method was performed. The effect of N-acylation and the position of basic aminoacids (Arg, Lys) in lipopeptides on antimicrobial activity was studied, and activity was expressed using MIC and IC_50_ (Table 2).

**Table 2:** Minimal inhibitory concentration of lipopeptides with C-terminal amidation against selected Gram-positive and Gram-negative bacteria and fungi

	**SHP-1.2**	SHP-2.2	SHP-3.2	**SHP-1.3**	SHP-2.3	SHP-3.3
MIC (µg/mL) / IC_50_ (µg/mL)						
*A. baumannii* DBM 3183	55.3/60.0	*N* / > 100.0	*N* / > 100.0	*N* / > 00.0	*N* / > 100.0	*N* / > 100.0
*E. coli* DBM 3001	78.9/100.0	*N* / > 100.0	68.7/100.0	*N* / > 100.0	*N* / > 100.0	*N* / > 100.0
*Ps. aeruginosa* DBM 3081	51.3/60.0	*N* / > 100.0	83.6 /100.0	*N* / > 100.0	*N* / > 100.0	*N* / > 100.0
*B. subtilis* DBM 3006	16.9/25.0	*N* / > 100.0	41.1/50.0	31.5/40.0	6.9/20.0	34.3/50.0
*E. faecalis* DBM 3075	32.6/40	*N* / > 100.0	*N* / > 100.0	71.5/100.0	*N* / > 100.0	*N* / > 100.0
*M. luteus* DBM 3053	**6.4/10.0**	**7.0/10.0**	**5.8/10.0**	**5.5/10.0**	**1.6/5.0**	**1.5/5.0**
*Staph. aureus* DBM 3002	27.7/50.0	*N* / > 100.0	8.6/30.0	**33.0/50.0**	**31.9/50.0**	**28.8/50.0**
*Staph. aureus* MRSA1	7.8/12.5	14.9/25.0	6.8/12.5	**30.6/50.0**	**25.1/50.0**	**25.2/50.0**
*C. tropicalis* DBM 2166	55.9/100.0	*N* / > 100.0	N / > 100.0	**10.3/30.0**	56.4/75.0	**10.0/30.0**

*N* value could not be determined

*DBM* collection of microorganisms of the Institute of Biochemistry and Microbiology UCT, Prague, *MIC* minimum inhibitory concentration, *IC50* half maximal inhibitory concentration

The initial screening of activity on the agar plates showed the zones of inhibition of microbial growth of all the tested compounds (data not presented). The next testing was performed using the microtitre dilution method. For screening activity, a concentration of *c* = 1 mg/mL was chosen. The primary hexapeptides with C-terminal amidation (SHP-1.1, SHP-2.1, and SHP-3.1) at such a concentration exhibited low activity against all the tested microorganisms. One hundred percent growth inhibition was only detected in three cases, namely, peptide SHP-1.1 against *Staphylococcus aureus* and *Micrococcus luteus* and peptide SHP-3.1 against *Bacillus subtilis.* In other cases, the growth inhibition was about 15% (data not shown). In general, the primary hexapeptides with C-terminal amidation were more active against Gram-positive bacteria and fungi. Lipopeptide conjugates with C-terminal amidation, e.g., PAL/LCA-peptides, exhibited 100% inhibition against all the tested microorganisms at a concentration equal to *c* = 1 mg/mL. PAL-peptides containing N-terminal palmitic acid were active against the widest spectrum of microorganisms. The MICs against Gram-positive bacteria were found in the concentration range *c* = 7–60 µg/ mL (antibiotics MICs range *c* = 2–25 µg/mL) (data not shown) and against fungi in the range *c* = 80–100 µg/mL (antimycotics MICs ≤ 2 µg/mL) (data not shown). The Gram-negative bacteria were less sensitive, and MICs under the breakpoint of 100 µg/mL were only determined for SHP-1.2 and the bacteria *Pseudomonas aeruginosa* (55 µg/mL, kanamycin 20 µg/mL) and *Acinetobacter baumannii* (60 µg/mL, tetracycline 10 µg/mL). LCA-peptides containing N-terminal lithocholic acid were slightly active against Gram-negative bacteria, highly active against Gram-positive bacteria with MICs in the range 7–50 µg/mL (antibiotics MICs range µg/mL) and also exhibited increased activity against fungi, MICs in the range of 30–60 µg/mL (antimycotics MICs ≤ µg/mL) (Table 2). Against *Staph. aureus* MRSA1, the LCA-peptides exhibited lower antibacterial activity with higher MIC values than PAL-peptides. The synthesized lipopeptides (PAL/LCA-peptides) were also subjected to antifungal screening against the yeast *Candida tropicalis*. The strongest effect against *Candida tropicalis* was observed for the LCA-peptides SHP-1.3 and SHP-3.3. The MIC value for SHP-1.3 was 10.3 µg/mL, and the MIC value for SHP 3.3 was 10.0 µg/mL.

## Characterization of bacteriostatic/bactericidal properties of PAL-peptide and LCA peptide

**Fig. 3:**
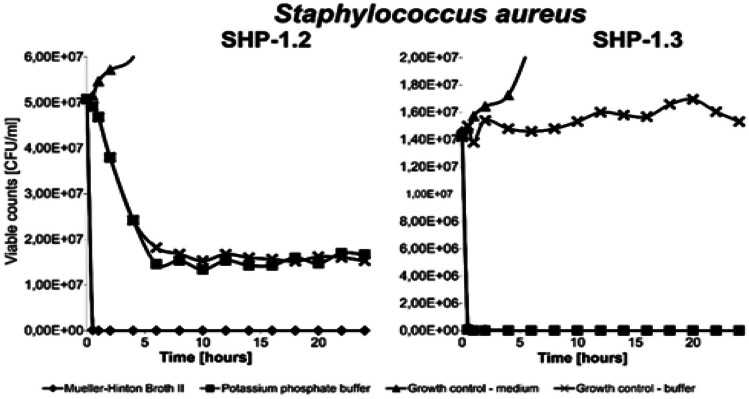
Antimicrobial effect of PAL/LCA-peptides SHP-1.2 and SHP-1.3 to bacteria *Staph. aureus* MRSA 1. Cell viability count of *Staph. aureus* MRSA1 with treatment with PAL/LCA peptides–lipopeptides SHP1.2 and SHP1.3

On the basis of previous results gained from the quantitative determination of MICs, we decided to only work with the PAL/LCA-peptides, mainly lipopeptide SHP-1.2 and lipopeptide SHP-1.3. Since the lipopeptide exhibited significant antimicrobial activity, our attention was further focused on monitoring the effect of this lipopeptide on the viability of the *Staphylococcus aureus* bacterial cultures. To evaluate whether the lipopeptide only inhibited growth (bacteriostatic) or killed the bacteria (bactericidal), lipopeptides were subjected to a bacterial killing experiment. This experiment was used to determine how quick the effect of PAL/LCA-peptides on bacteria is and whether the activity of these compounds is dependent on the presence of active bacterial metabolism (Kamysz et al. 2007). Two different media, one with nutrients and the second without, were chosen for estimating the mechanism of action. We first investigated the kinetics of the killing potential of PAL/LCA-peptides by incubating approximately 10^7^ bacterial cells (OD_600_ 0.1) with each of the PAL/LCA-peptides. SHP-1.2 and SHP-1.3 were able to kill 100% of bacteria within half an hour (Fig. 3), SHP-1.2 only in medium with nutrients and SHP-1.3 in both media. This may show the difference in their mechanism of action, specifically that SHP-1.2 could act in a metabolic pathway and SHP-1.3 as a membrane destabilizing agent, because its action seems to be independent of the metabolism. The killing kinetics of these PAL/LCA-peptides was independent of cell envelope composition, and both sensitive Gram-positive and Gram-negative bacteria responded similarly to the action of the peptides.

In parallel with this experiment, we investigated the proportion of cells with a damaged bacterial membrane after 1 h incubation with PAL/LCA-peptide lipopeptides. Disruption of the microbial membrane (membrane damage) was followed by the detection of lactate dehydrogenase activity, and two different media, one with nutrients and the second without, were chosen for estimating the mechanism of action again. The comparison of results of killing potential and damage to the membrane was not so clear. It was confirmed that the membrane disruption involved in the mechanism of action is only one possible factor. Mostly, only 30% of bacterial cells were damaged, even if no viable cell was found **(**Table 3).

**Table 3:** Comparison of effect of N-acylation of lipopeptide SHP-1.2 and SHP-1.3 on cell membrane damage and killing activity

**SHP-1.2 ( PAL lipopeptide)**				**SHP-1.3 (LCA lipopeptide)**			
**killing activity (%)**		**Membrane damage (%)**		**killing activity (%)**		**Membrane damage (%)**	
MHB	KP	MHB	KP	MHB	KP	MHB	KP
100 ± 15	8 ± 1	26 ± 3	32 ± 4	100 ± 12	100 ± 18	0 ± 5	30 ± 5

*MHB* Mueller–Hinton broth II, *PP* potassium phosphate buffer

## DNA binding properties of lipopeptides with C-terminal amidation

According to the previous results, we decided to clarify one of the possible molecular interaction mechanisms, the ability of the studied lipopeptides to bind microbial plasmid DNA. Based on the assumption that pore formation is only one of the influencing factors, which arose from previous results, the effect on one of the possible intracellular targets, DNA, was determined. This determination was carried out on isolated plasmid DNA, specifically on two plasmids, pGreen 0029 and pBluescript II KS- in circular and linearized form.

**Fig. 4:**
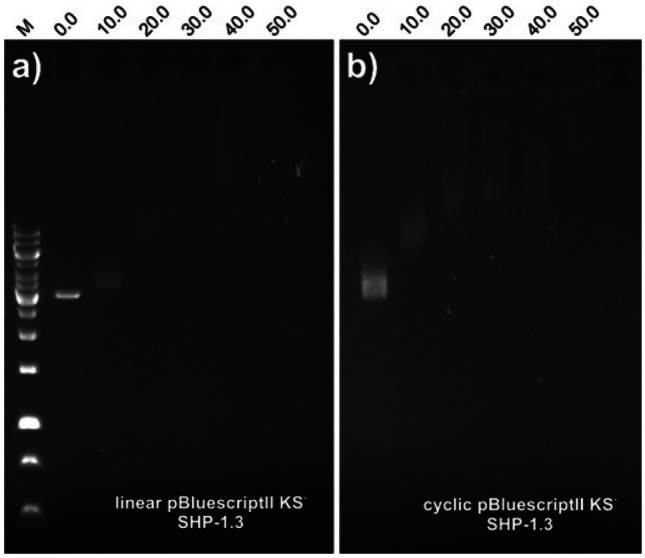
The inhibition effect of lipopeptide SHP-1.3 on the migration of both circular and linearized plasmid DNA (pBluescriptII KS-). Various amounts of lipopeptide were incubated with 50 ng of circular (**a**) or linearized (**b**) plasmid DNA at room temperature for 1 h, and the reaction mixtures were applied to a 1% agarose gel. The weight ratios (peptide:DNA) are indicated above each lane

This mechanism was clarified for the antimicrobial peptides buforin II and magainin 2 (Park et al. 1998). The ability to bind DNA was examined by a comparison of the electrophoretic mobility of circular or linearized plasmid DNA bands at various weight ratios of PAL/LCA-peptides on 1% agarose gel. We found that all of the tested PAL/LCA-peptides were able to bind both circular and linearized DNA (Fig. 4).

The highest ability to influence the electrophoretic mobility of the circular and linearized plasmid pGreen 0029 was exhibited by lipopeptide SHP-3.3 in the ratio of 50 ng lipopeptide:50 ng circular DNA (1.0) and 500 ng lipopeptide:50 ng linear DNA (10.0). With plasmid pBluescript II KS-, the highest affinity was recorded for lipopeptide SHP-2.3 in ratios of 10.0 and 10.0, respectively. Only lipopeptides SHP-1.2 and SHP-3.2 in the tested ratios did not exhibit any influence on the electrophoretic mobilities of circular or linearized forms of plasmid pGreen 0029.

## Effect of lipopeptides with C-terminal amidation on morphological changes of bacteria

**Fig. 5:**
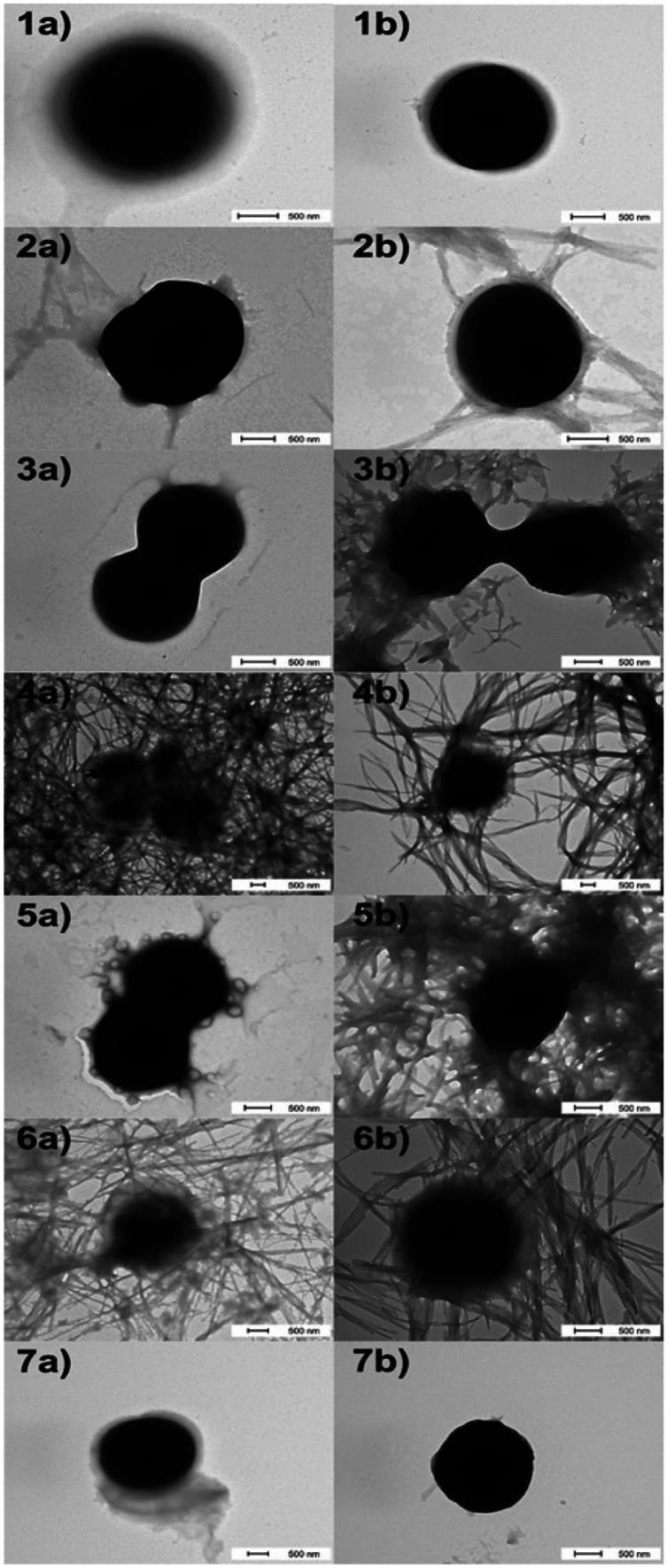
Visualization of cells of *Staph. aureus* untreated (1a/b) and treated with lipopeptides SHP-1.2 (2a/b), SHP-1.3 (3a/b), SHP-2.2 (4a/b), SHP-2.3 (5a/b), SHP-3.2 (6a/b), and SHP-3.3 (7a/b). Pictures were obtained by transmission electron microscopy without staining. Bacteria were incubated with lipopeptides for 1 to 4 h in two different media, with nutrients (Mueller–Hinton broth 2) (**a**) or without nutrients (potassium phosphate buffer – 1 M, pH 7) (**b**). Dash 500 nm

Time-dependent transmission electron microscopy (TEM) studies were performed to see the effect of PAL/LCA peptides on the architecture of *Staph. aureus*. The effect of all lipopeptides was visualized using transmission electron microscopy after 1 to 4 h of incubation of bacterial cells with each PAL/LCA-peptide in both media, with and without nutrients, respectively. Some PAL/LCA-peptides seem to act via damaging cellular envelopes (capsule, wall, or membrane). This effect is observable for SHP-1.3 and SHP-2.3 in medium with nutrients and SHP-3.3 in both media. In the remaining cases, the formation of fibrous bodies and gradual destruction of the cells was observed (Fig. 5). Based on transmission electron microscopy, the mechanism of antibacterial action could be a combination of membrane damage caused by the binding of synthetic lipopeptides to cell envelopes and their subsequent aggregation into fibers.

## Cytotoxicity

One of the most desired properties of AMPs is low toxicity to eukaryotic cells. We assessed the cytotoxicity of the lipopeptides by testing their ability to lyse sheep red blood cells. Hemolytic activity was determined in serial dilutions from the MIC value to a concentration equal to 100 µg/mL of (PAL/LCA)-peptides. The hemolytic peptide chrysophsin-3 at a concentration equal to *c* = 10 µg/mL was used as the positive control. The negative control was the solvent, deionized water. No zones of hemolysis of SHPs were detected at any of the tested concentrations. Further, we investigated the toxic effect against mammalian cells by incubating the human liver hepatocellular carcinoma cell line HepG2 ATCC^®^HB-8065TM or human embryonic kidney cell line HEK293T ATCC^®^CLR-11268TM with each PAL/LCA-peptide. Toxicity was determined in a serial dilution, and the applied concentrations of PAL/LCA-peptides were 50, 100, and 200 µg/mL. The kidney cells were significantly more sensitive than the liver cells. The inhibition effect of all tested PAL/LCA-peptides on kidney cells was mostly approximately about 30% higher than that on liver cells. PAL/LCA-peptides SHP-2.3 and SHP-3.3 exhibited the lowest toxicity against human liver cells, their inhibition effect was at most 5% for the highest concentration, and the growth inhibition was mostly equal to 50% for 50 µg/mL, 70% for 100 µg/mL, and 85% for 200 µg/mL. Peptide SHP 1.3 had a high efficiency on HepG2 cells (97%) (Table 4).

**Table 4:** Inhibitory effect of synthetic lipopeptides and chrysophsin-3 on tissue cultures of HepG2 human liver tumor cells and HEK263T human kidney embryonic cells after 24 h of exposure to the tested substance

		**HepG2**	**HEK263T**
	c (µg.ml^−1^)	% inhibition	% inhibition
**SHP-1.2**	50	4.1 ± 0.6	37.6 ± 5.6
	100	42.2 ± 6.3	77.8 ± 11.7
	200	67.9 ± 10.2	73.1 ± 11.0
**SHP-2.2**	50	4.3 ± 0.6	11.1 ± 1.7
	100	11.4 ± 1.7	19.3 ± 2.9
	200	18.5 ± 2.8	28.6 ± 4.3
**SHP-3.2**	50	17.0 ± 2.6	4.1 ± 0.6
	100	20.7 ± 3.1	29.6 ± 4.4
	200	23.2 ± 3.5	0.0 ± 0.3
**SHP-1.3**	50	57.4 ± 8.6	63.8 ± 9.6
	100	**92.8 ± 13.9**	72.0 ± 10.8
	200	**97.8 ± 14.7**	84.1 ± 12.6
**SHP-2.3**	50	7.4 ± 1.1	25.6 ± 3.8
	100	0.0 ± 2.2	42.2 ± 6.3
	200	23.7 ± 3.6	85.1 ± 12.8
**SHP-3.3**	50	4.3 ± 0.6	7.8 ± 1.2
	100	35.6 ± 5.3	28.5 ± 4.3
	200	44.6 ± 6.7	88.2 ± 13.2
**Chrysophsin-3**	10	5.9 ± 0.9	7.9 ± 1.2
	50	58.8 ± 8.8	64.8 ± 9.8
	100	94.6 ± 14.2	98.6 ± 14.8

## Discussion

The majority of the known antimicrobial peptides have been isolated from natural sources. Recently genomic and proteomic methods have also been applied for peptide identification. Knowledge obtained by both classical and novel methods can be used to design the chemical synthesis of new peptide compounds with biological properties. The sequence analysis of natural antimicrobial peptides led to a template, with which it is possible to generate active AMPs (Tossi et al. 1997; Giangaspero et al. 2001; Tossi et al. 2000). We designed three scrambled hexapeptides, one according to this template, and the other two with a random sequence. Each of these peptides was synthesized with C-terminal amidation (Mekler 1994), and subsequently a fatty or steroidal acid was joined to the N-terminus (Radzishevsky et al. 2005). Cationic peptide antibiotics (CPAs), also termed cationic antimicrobial peptides, have been isolated from organisms ranging from bacteria to mammals. In general, CPAs can be considered to be facially amphiphilic (Savage et al. 2002). Two of the major classes of CPAs are β-sheet-forming and α-helix-forming peptides. In both classes of compounds, side chains from hydrophobic residues are segregated on one face of the molecule, while cationic side chains are oriented on the other face. Cationic steroid antibiotics (CSAs) have proven to have antibacterial activities similar to those of many CPAs. Cholic acid (CA) is a naturally occurring facial amphiphile that mimics the amphiphilic nature of AMPs. Numerous CA-derived amphiphiles have been engineered for antimicrobial activity, and it has been shown that these amphiphiles, similar to AMPs, interact with bacterial membranes and cause membrane disruptions (Gupta et al. 2019). Based on our results, it is evident that the template sequence (SHP-3.x) was not the most effective against microorganisms, compared with the other two sequences, but it was less toxic. Random (PAL/LCA)-peptides SHP-1.x and the lowest (PAL/LCA)-peptides SHP-2.x exhibited the highest biological activities. The main difference in sequences was in the position of the cationic amino acids arginine and lysine. In SHP-1.x and SHP-3.x, these amino acids were located close one to another, positions 2, 3, or 4, 5. In SHP-2.x, the cationic amino acids were separated, positions 3 and 6. The template sequence was also unable to completely destroy the bacterial cells. It seems to act via an interaction with a metabolic pathway, not with the membrane, because the action of this peptide was dependent on the active metabolism of the target microorganism (Meylaers et al. 2003; Kamysz et al. 2007). This finding could explain the low level of toxicity against mammalian cells. The random sequences were able to kill all of the bacteria completely in 0.5 or 2 h. Their mechanism of action was their destructive effect on the membrane. However, none of our experiments on the mechanism of action gave clear results. Instead, it is likely that many factors act together, and one of them damages membranes. One of these factors seems to be able to bind DNA. Recently, it has been reported that some polymeric cationic antimicrobials could enter into the bacterial cytoplasm and combine with DNA to quickly generate reactive oxygen species (ROS) to kill bacteria (Lin et al. 2021).We tested this mechanism by analyzing the electrophoretic mobility of circular and linearized plasmid DNA in 1% agarose gel. Based on our results, we suppose that the interaction between these peptides and DNA is partially specific, because we were able to report a shift of bands, but in some cases, this effect was not as clear and the bands expanded into broad zones, which may mean the occurrence of an electrostatic interaction. Also, all of the LCA-peptides were able to bind both types of DNA more tightly than PAL-peptides; the tight binding of LCA-peptides could be reinforced by the interaction between DNA and cyclic structures of LCA itself. The effect of a fatty or steroidal acid joining the structure was demonstrated by the rapid increase in antimicrobial activity, but the spectrum and the rate of activity were more dependent on the sequence of the basic peptide. The effect of acyl conjugation on the biological properties of peptides is known (Radzishevsky et al. 2005; Nair 1988). We established a hypothesis that joining the steroidal acid to the peptide molecule could increase the activity of the peptide against eukaryotic cells. The LCA-peptides exhibited increased activity against *Candida* species and higher toxicity against mammalian cells. Some steroids and bile acids, such as that of LCA, demonstrated a wide range of potentially toxic biological activities; some of these activities are mutagenic, transforming, or DNA-strand breakage (Venturi et al. 1997). The direct effect of LCA on hepatocytes, such as with obstructive cholestasis, can induce inflammatory genes and cause damage (Nair 1988). This finding could be helpful for understanding the in vitro hepatotoxicity of LCA-peptides. LCA was also reported to be a mutagenic factor in the concentration range 200–400 µM (0.075–0.15 µg/mL). LCA precipitated from the solution at higher concentrations than 400 µM and lost its DNA-damaging effects (Ball et al. 2004). Using transmission electron microscopy, we tried to approximate the mechanism of action in a better way. Fibrous bodies are visible in almost every captured picture, except for the effect of SHP-3.3. This could be explained by the ability of lipopeptides to aggregate (Shai 2002; Majerle et al. 2003; Zanetti et al. 1995). Enhanced binding activities and aggregation of lipopeptides could cause the packaging of cells and their slower destruction. This theory and results from LDH determination are useful for explaining our finding that no viable cells were found after treatment with PAL/LCA-peptides, but only 30% of cells were damaged. Also, with the LCA-peptides, binding to the Fas receptor and hence triggering of the apoptosis cascade are possible (Korzeniewski and Callewaert 1983).

In this study, for the first time, we demonstrated a comparison of the positive effects of N-terminal substitution by palmitic acid and litocholic acid into the molecule of synthetic short lipopeptides with an amidated C–terminus on their antibacterial and antifungal activity. In this work, we described some biological properties of short lipopeptides and their changes resulting from amino acid sequence modifications. At first, we designed and synthesized three scrambled hexapeptides with the required properties. We found that the basic peptides are almost ineffective. To increase their biological activities, the N-terminal binding of an acyl group was designed, and lipopeptides were prepared. There were two types of lipopeptides, the former one with palmitic acid and the latter one with lithocholic acid. Both types of lipopeptides were highly active against Gram-positive bacteria with MIC values under 100 µg/mL. PAL-peptides exhibited activity against a wider spectrum of microorganisms, including Gram-negative bacteria and fungi. LCA-peptides exhibited good activity against Gram-positive bacteria and increased activity against fungi, especially against *Candida* species. The sequences were designed either randomly or according to the template. The randomly designed sequences of SHP1.3 were generally more active, and both their antimicrobial and activities on HepG2 human liver hepatocellular carcinoma cells were higher. The template sequence gave a slightly reduced antimicrobial activity but greatly reduced toxicity. The position of the cationic amino acid is important for activity, especially that of Lys and Arg; the best results were achieved with lipopeptide SHP-1.3 – LCA-LV**KR**AG-NH_2_, bearing those two amino acids in close proximity.

## Data Availability

The datasets generated during and/or analyzed during the current study are available from the corresponding author on reasonable request.
